# Gene expression profile in functioning and non-functioning nodules of autonomous multinodular goiter from an area of iodine deficiency: unexpected common characteristics between the two entities

**DOI:** 10.1007/s40618-021-01660-y

**Published:** 2021-08-17

**Authors:** P. Agretti, G. De Marco, E. Ferrarini, C. Di Cosmo, L. Montanelli, B. Bagattini, L. Chiovato, M. Tonacchera

**Affiliations:** 1 Endocrinology Unit 1, University Hospital of Pisa, Via Paradisa 2, 56124 Pisa, Italy; 2 Department of Clinical and Experimental Medicine, Section of Endocrinology, University of Pisa, Via Paradisa 2, 56124 Pisa, Italy; 3 Unit of Internal Medicine and Endocrinology, Laboratory for Endocrine Disruptors, IRCCS Maugeri, 27100 Pavia, Italy; 4 Department of Internal Medicine and Therapeutics, University of Pavia, 27100 Pavia, Italy

**Keywords:** Gene expression, Thyroid nodule, Autonomous multinodular goiter

## Abstract

**Purpose:**

Toxic multinodular goiter is a heterogeneous disease associated with hyperthyroidism frequently detected in areas with deficient iodine intake, and functioning and non-functioning nodules, characterized by increased proliferation but opposite functional activity, may coexist in the same gland. To understand the distinct molecular pathology of each entity present in the same gland, the gene expression profile was evaluated by using the Affymetrix technology.

**Methods:**

Total RNA was extracted from nodular and healthy tissues of two patients and double-strand cDNA was synthesized. Biotinylated cRNA was obtained and, after chemical fragmentation, was hybridized on U133A and B arrays. Each array was stained and the acquired images were analyzed to obtain the expression levels of the transcripts. Both functioning and non-functioning nodules were compared versus healthy tissue of the corresponding patient.

**Results:**

About 16% of genes were modulated in functioning nodules, while in non-functioning nodules only 9% of genes were modulated with respect to the healthy tissue. In functioning nodules of both patients and up-regulation of cyclin D1 and cyclin-dependent kinase inhibitor 1 was observed, suggesting the presence of a possible feedback control of proliferation. Complement components C1s, C7 and C3 were down-regulated in both types of nodules, suggesting a silencing of the innate immune response. Cellular fibronectin precursor was up-regulated in both functioning nodules suggesting a possible increase of endothelial cells. Finally, Frizzled-1 was down-regulated only in functioning nodules, suggesting a role of Wnt signaling pathway in the proliferation and differentiation of these tumors. None of the thyroid-specific gene was deregulated in microarray analysis.

**Conclusion:**

In conclusion, the main finding from our data is a similar modulation for both kinds of nodules in genes possibly implicated in thyroid growth.

## Introduction

Autonomous or toxic multinodular goiter is a heterogeneous disease-producing hyperthyroidism frequently found in iodine-deficient areas [1]. The term “autonomous” multinodular goiter (AMNG) or “toxic” multinodular goiter (TMNG) encompasses a spectrum of different clinical entities, ranging from a single hyperfunctioning nodule within an enlarged thyroid that also contains non-functioning nodules to multiple hyperfunctioning areas scattered throughout the gland, barely distinguishable from non-functioning nodules and extranodular parenchyma [2, 3]. Thyroid scintiscan after the administration of tracer doses of radioactive iodine or 99mTc can classify thyroid nodules as functioning and non-functioning [1–4]. While functioning nodules are able to trap radioiodine and are also defined “hot” nodules, non-functioning nodules are those that, compared with the normal thyroid tissue, take up little or no radioiodine and, for this peculiarity, assume the typical “cold” appearance at thyroid scintiscan. Thyroid nodules of TMNG or AMNG may be true adenomas, defined by the presence of a well-formed fibrous capsule, or more commonly hyperplastic lesions consisting of aggregates of micro/macro follicles lacking encapsulation and not clearly delimited from the surrounding parenchyma [4]. The development of nodular goiter is a result of long-term exposure of the thyroid gland to proliferative stimuli, such as iodine deficiency, goitrogen substances or congenital errors in thyroid hormone synthesis, resulting in insufficient thyroid hormone production and stimulation of TSH secretion by the pituitary. TSH determines a short-term upregulation of iodine uptake and organification, thyroglobulin synthesis and T3 and T4 secretion, and a long-term proliferation of follicular cells with enlargement of the thyroid gland [3].

Pivotal studies showed that up to 82% of solitary toxic adenomas harbor activating TSH receptor (TSHr) mutations [5–7]. Subsequent studies reported that activating TSHr mutations are not only present in most solitary toxic adenomas, but also in hyperfunctioning areas (either adenomas or hyperplastic nodules) within a toxic multinodular goiter [8, 9]. In particular, somatic mutations determining the constitutive activation of TSHr are reported in about 60% of functioning nodules, while the residual 40% do not harbor TSHr mutations and the genetic mechanism behind remains poorly understood. On the other hand, while a defective iodine transport and organification are implicated in hypofunctionality of non-functioning nodules, the molecular event accounting for the proliferative advantage in these nodules is poorly understood [10]. No specific genetic mutation has been described so far in non-functioning nodules, however, a minority of these nodules harbor gene mutations that are also common to some malignant follicular neoplasms (N-RAS, H-RAS, K-RAS mutations or RET rearrangements) [10].

Thyroid tumors result from changes in gene expression patterns that are important for cellular regulatory processes such as growth, differentiation, DNA duplication, mismatch repair and apoptosis. Classification of human tumors into distinct groups based on their origin and histopathological appearance has been the foundation for diagnosis and treatment. This classification is generally based on cellular architecture, and cell-specific antigens only. In contrast, gene expression assays have the potential to identify thousands of unique characteristics for each tumor type [11]. For this reason, we decided to compare the gene expression profile of healthy tissue and functioning and non-functioning thyroid nodules arising in the same gland of patients with AMNG.

## Patients and methods

### In vitro tests

Serum measurement of thyroid parameters was performed in all patients included in the study. FT4 and FT3 were measured by chemiluminescent immunoassay (Vitros System, Ortho-Clinical Diagnostic, Rochester, NY, USA). Thyrotropin was assessed by a sensitive chemiluminescence assay (Immulite 2000, DPC, Los Angeles, CA, USA).

Anti-thyroglobulin (AbTG) and anti-thyroperoxidase (AbTPO) antibodies were measured using a two-step immunoenzymatic assay (AIA-Pack TgAb and TPOAb; Tosoh, Tokyo, Japan). The presence of antibodies directed against the TSH receptor (TRAB) was investigated by using a commercial radioreceptor assay (TRAK assay, B.R.A.H.M.S., Berlin, Germany).

### Patients

Two patients subjected to near-total thyroidectomy for AMNG were included in the study. At diagnosis, the patients showed subclinical hyperthyroidism with normal serum FT4 and FT3 and undetectable TSH. No autoimmunity signs were present in both patients with negative AbTG, AbTPO and TRAB. Physical examination, ultrasound, scintiscan imaging using ^131^I and histological examination were used to study thyroid glands.

Surgical tissue specimens were carefully dissected, matching the scintiscan with the whole gland laid on the pathologist tray in its proper anatomic orientation. Functioning and non-functioning nodules identified by scintiscan and healthy normal tissue were isolated and used for histological examination and molecular studies.

### Total RNA isolation

Tissue specimens were frozen in liquid nitrogen and stored at –80° C until processed for RNA isolation. Total RNA was isolated from approximately 30–40 mg of frozen tissue using TRIzol reagent (Invitrogen, Carlsbad, CA) according to the manufacturer's instructions. The quality of RNA samples was assessed by electrophoresis through denaturing agarose gels and staining with ethidium bromide to visualize the 18S and 28S RNA bands under UV illumination. The extraction yield was quantified spectrometrically at 260 nm.

### Double strand cDNA synthesis

To produce single-strand cDNA, five micrograms of total RNA for each sample were reverse transcribed for 1 h at 42 °C in a 20 μl reaction volume using 200 units of Superscript II Rnase H^−^ reverse transcriptase (Invitrogen, Carlsbad, CA) in the presence of 5 μM GeneChip T7-Oligo(dT) Promoter Primer (Affymetrix, Santa Clara, CA). Double-stranded cDNA was synthesized at 16° C by adding 10 units DNA Ligase, 40 units DNA Polymerase I and 10 units T4 DNA Polymerase (Invitrogen, Carlsbad, CA).

### In vitro transcription and microarray analysis

Biotin-labeled cRNA was prepared by in vitro transcription using BioArray High Yield RNA Transcript Labeling Kit (ENZO Life Sciences, Inc., Farmingdale, NY) following the standard Affymetrix protocols. Biotinylated cRNAs were cleanup, quantified and 15 μg were fragmented and added to a hybridization cocktail according to Affymetrix procedures.

After quality determination on Test-3 arrays, the samples were hybridized using the GeneChip Hybridization Oven 640 (Affymetrix, Santa Clara, CA) for 16 h at 45 °C to GeneChip Human Genome U133 set (HG-U133A and HG-U133B). The arrays were washed and stained with streptavidin–phycoerythrin using the Affymetrix antibody amplification protocol for eukaryotic targets (EukGE-WS2 protocol) in the GeneChip Fluidics Station 400 (Affymetrix, Santa Clara, CA). Images were acquired using the GeneArray Scanner (Agilent Technologies, Palo, CA), and row data were collected and analyzed by using the Affymetrix Micro Array Suite (MAS) version 5.0 software.

### Gene expression analysis

MAS 5 absolute and comparison expression analysis were performed according to Affymetrix GeneChip Expression Analysis Technical Manual’s protocols. An absolute expression analysis determines whether the transcripts represented on the probe array are present, absent or marginal in the sample. The comparison analysis identifies relative changes in the expression level of each transcript represented on the arrays. To compare the signals of several arrays, each experiment has to be scaled to the same target intensity (100) to take into account the inherent differences between the arrays and their hybridization efficiencies. Comparisons were made between the functioning and non-functioning nodules and the healthy non-nodular tissue of each patient on HG-U133A and HG-U133B, using as baseline the values obtained for the healthy non-nodular tissue. The output is a change call of increase, marginal increase, decrease, marginal decrease or no change, a p-value associated with the change call, and the intensity of the difference (signal log ratio).

### Linking the expression data to biological pathways

DNA microarray experiments simultaneously measure the expression levels of thousands of genes, generating huge amounts of data. We used GenMAPP (Gene MicroArray Pathway Profiler, downloaded from www.GenMAPP.org) program for viewing and analyzing gene expression data in the context of known biological pathways [12, 13].

Excel file of MAS obtained gene expression data, including probe set ID, signal log ratio (SLR), SLR low and high, change call and change *p* value. Expression data were imported into GenMAPP in a csv format (comma separated values) and converted into a gene expression data set that can be viewed on the different MAPPs with specific color codes. When a MAPP is linked to a gene expression data set, GenMAPP automatically and dynamically color codes the genes on the MAPP based on data and criteria provided by the investigator. In this paper, we defined the following color codes: red corresponding to strong increase (SLR ≥ 1.5 and change = increase), pink corresponding to valid increase (SLR low ≥ 0.1 and *p* value ≤ 0.04), blue corresponding to strong decrease (SLR ≤ -1.5 and change = decrease), sky-blue corresponding to valid decrease (SLR ≤ -0.1 and *p* value ≥ 0.996).

### Reverse transcription

One microgram of total RNA for each sample was reverse transcribed for 1 h at 42 °C in a 20 μl reaction volume using 200 units of Superscript II Rnase H^−^ reverse transcriptase (Invitrogen Life Technologies, Carlsbad, CA, USA) in the presence of 1.5 μM random examers (Pharmacia Biotech, Uppsala, Sweden), 0.01 M DTT and 1 mM dNTP mix.

### Determination of mRNA levels by using real-time RT-PCR

Quantitative gene expression studies were performed using TaqMan Gene Expression Assays pre-designed primer and probe sets (Applied Biosystems, Foster City, CA). PCR reaction was carried out in 96-well optical reaction plates using a cDNA equivalent of 0.5 ng total RNA for each sample in a volume of 50 μl using the TaqMan Universal PCR Master Mix (Applied Biosystems, Foster City, CA) according to the manufacturer's instructions. PCR was developed on the ABI Prism 7700 Sequence Detector (Applied Biosystems, Foster City, CA). The thermal cycling conditions comprised an initial denaturation step at 95 °C for 10 min and 40 cycles of two-step PCR, including 15 s of denaturation at 95 °C and 1 min of annealing-elongation at 60 °C, using the standard protocol of the manufacturer. Each sample was assayed in triplicate and the intra-assay coefficient of variation was less than 1%. Experiments were repeated three times. The monitoring of negative controls for each target showed an absence of carryover.

To minimize the errors arising from the variation in the amount of starting RNA among samples, amplification of β-actin mRNA was performed as an internal reference against which other RNA values can be normalized. The primers and the probe for the β-actin RNA were purchased from Applied Biosystems, Foster City, CA and the amplification was started from 0.5 ng total RNA.

Normalized results were expressed as the ratio of the pg RNA of the target gene to the pg RNA of the β-actin gene (mean ± SE of three experiments).

## Results

### Gene expression analysis

For each human array containing about 22,000 transcripts (probe sets) we deleted all the genes with a call no change, and we selected all the increased genes with a signal log ratio ≥ 1 (2-fold increased with respect to the baseline) and all decreased genes with a signal log ratio ≤ − 1 (2-fold decrease with respect to the baseline). In Table 1 the percentage of changed genes selected for the functioning and non-functioning nodules with respect to the non-nodular healthy tissue in patient #1 and #2 on HG-U133A and B are shown. In both patients, we observed a major number of regulated genes (up-regulated or down-regulated) in the functioning nodules with respect to the non-functioning ones. In particular, the number of increased genes in the functioning nodule of patient #2 was about 5-fold greater with respect to the non-functioning nodule of the same patient (Table 1).

**Table 1:** Percent of modulated genes in functioning nodule (FN) and non-functioning nodule (NN) of patient #1 and #2

	Patient #1		Patient #2	
	FN	NN	FN	NN
% Increase	8.9	5.4	6.0	1.3
% Decrease	8.4	6.5	8.9	5.3
% Total change	17.3	11.9	14.9	6.6

The software GenMAPP allowed us to introduce selected genes in the context of known biological pathways. In Table 2, 29 metabolic pathways inside of which the software placed a total of 109 selected genes with their name, GeneBank accession number, chromosome localization and signal log ratio relative value are shown.

### Linking the expression data to biological pathways

**Fig. 1:**
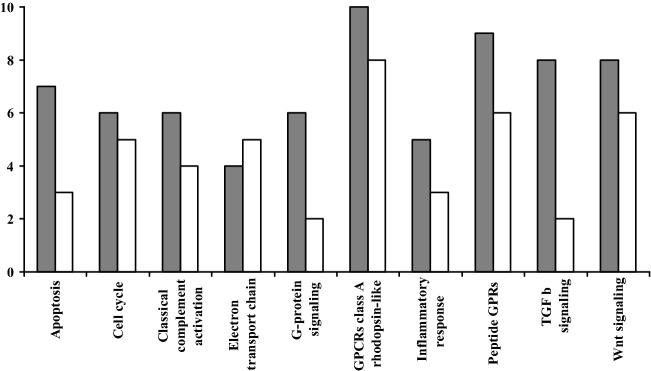
Number of regulated genes in functioning nodules (FN, grey bars) and non-functioning nodules (NN, white bars) belonging to ten metabolic pathways prominently involved

**Fig. 2:**
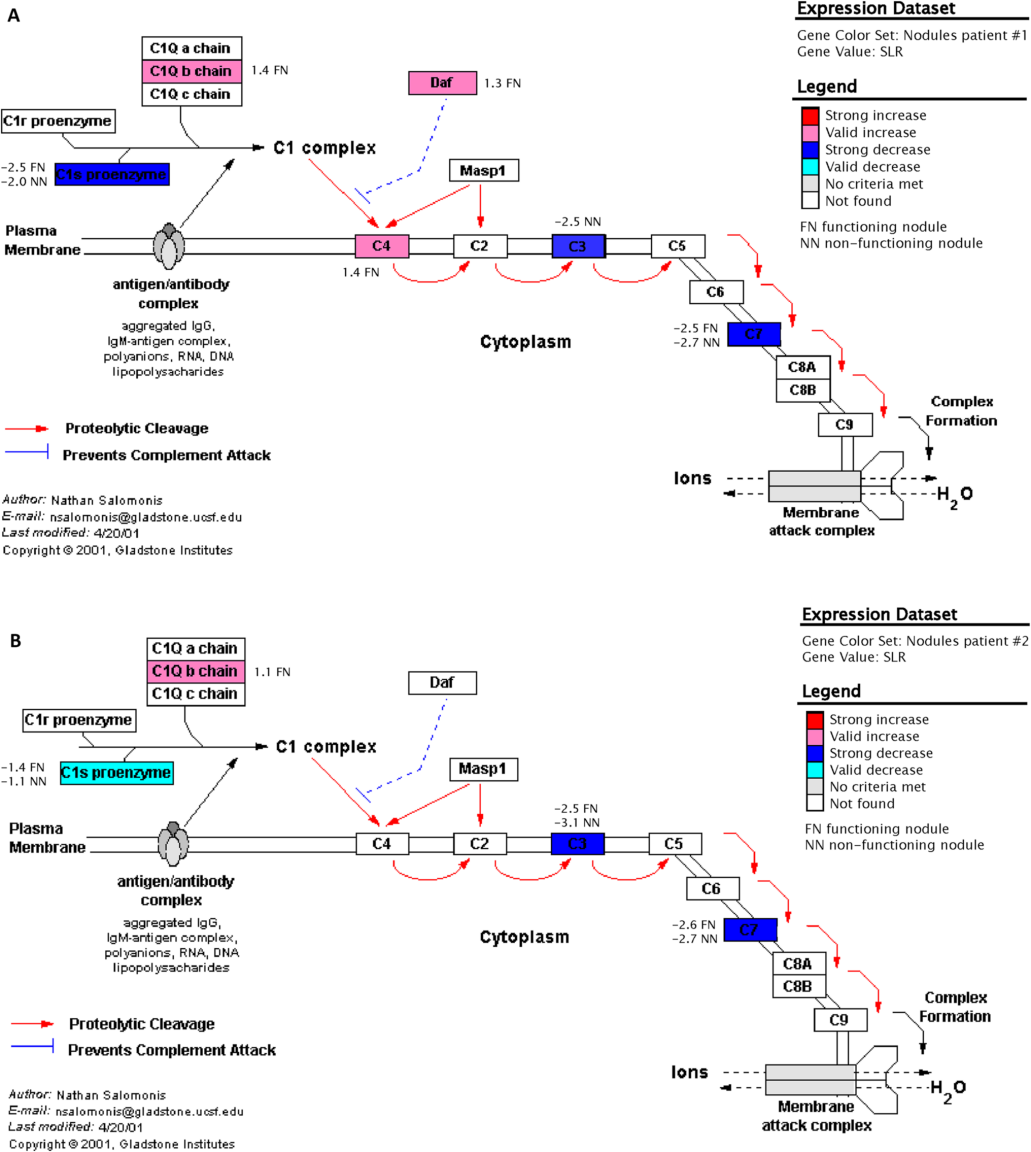
Diagram of the classical complement activation pathway adapted from a view in GenMAPP in functioning and non-functioning nodules of patient #1 (**A**) and patient #2 (**B**); each box represents a gene. The colour codes are described in the text

Data analysis prominently revealed a regulation in the expression of genes associated with apoptosis (8 genes), cell cycle (9 genes), classical complement activation (6 genes), electron transport chain (7 genes), inflammatory response (5 genes), GPCRs class A rhodopsin-like (13 genes), G-protein signaling (7 genes), peptide GPCRs (10 genes), TGF β signaling (9 genes) and Wnt signaling (11 genes) in functioning and non-functioning nodules of the two patients. In all the above biological pathways we observed a major number of regulated genes in functioning nodules with respect to the non-functioning ones (Fig. 1). The classical complement activation pathway was the one with the greater number of genes which were simultaneously up- or down-regulated in both functioning and non-functioning nodules (Fig. 2A, B).

### Chromosomal localization of up- and down-regulated genes

When analyzing the regulated genes in functioning and non-functioning nodules from patient #1 and #2 for their chromosomal location, it became apparent that multiple chromosomes were more frequently involved. When corrected for chromosome size and each chromosome’s individual total predicted gene content, the regulated genes in functioning and non-functioning nodules from the two patients were most frequently found on chromosome 1, 2, 4, 5, 6, 12, 16, 18, and 22. Moreover, the chromosomes 1, 12, 16, 19 and 22 displayed the highest relative distribution of genes per Mbase-pair of chromosome size. In functioning nodules of both patients regulated genes were more frequently found on chromosome 1, 3, 4, 6, 9, 12, 16, 19 and 22 with the highest distribution on chromosome 19, while in non-functioning nodules of the same patients modulated genes were preferentially located on chromosomes 1, 2, 5, 6, 12, 14, 18 and 20 with the highest distribution on chromosome 1.

### Up-regulated genes

The cellular fibronectin precursor was the gene with the strongest increase of expression (signal log ratio 3.4) in the functioning nodule of patient #1 (Table 2).

**Table 2:** Results of gene array studies: selected genes with their name, gene bank accession number, chromosome localization and signal log ratio relative value, are shown

Biological pathway	Gene Bank accession number	Chromosome location	Patient #1		Patient #2	
			≥ 2-fold changes		≥ 2-fold changes	
			FN	NN	FN	NN
**Apoptosis**						
TNF-related apoptosis inducing ligand (TRAIL)	U37518	**3**q26.31	− 1.3			
Tumor necrosis factor receptor type 1 (TNFR1)	X55313	**12**p13.31	1.0			
Bcl2	M14745	**18**q21.33	− 1.3			
BH3 interacting domain death agonist (BID)	AF042083	**22**q11.21	2.7			
Cytochrome c (Cytc)	BC005299	**7**p15.3		1.2		
IAP 1	AF070674	**11**q22.1			− 1.8	− 2.0
c-Jun	J04111	**1**p32.2			− 1.9	− 1.8
TRAF2	U12597	**9**q34.3			1.6	
**Blood clotting cascade**						
von Willebrand factor	M25828	**12**p13.31		1.6		
Coagulation factor V	M14335	**1**q24.2		1.3		
Pro-urokinase plasminogen activator	M15476	**10**q22.2	1.6			
**Calcium channels**						
Inositol 1,4,5-trisphosphate receptor type 1 (IP3R1)	D26070	**3**p26.1	− 2.0		2.1	
Ryanodine receptor 1 (RYR1)	U48508	**19**q13.13	2.4			
**Cell cycle**						
Cyclin-dependent kinase inhibitor 1 (Melanoma differentiation associated protein 6) (Cip1)	U09579	**6**p21.31	1.3	1.3	1.6	1.0
Transforming growth factor beta 1 precursor (TGF-β1)	BC000125	**19**q13.2	1.4			
Cyclin D3 (CycD3)	M92287	**6**p21.1	1.3			
Cyclin D2 (CycD2)	D13639	**12**p13.32	1.0			
Histone deacetylase 4 (HDAC4)	AB006626	**2**q37.3	− 1.0			
P107 (RBL1)	AL136172	**20**q11.23			− 1.1	
SMAD4 (DPC4)	AF045438	**18**q21.1				1.1
RB1	M27845	**13**q14.2				1.0
PTTG3	AF095289	**5**q33.3		1.0		
**Classical complement activation**						
Complement component 1, β-chain (C1q β)	X03084	**1**p36.12	1.4		1.1	
Decay-accelerating factor (DAF)	M31516	**1**q32.2	1.3			
Complement subcomponent C1s, α- and β-chains (C1s)	M18767	**12**p13.31	− 2.5	− 2.0	− 1.4	− 1.1
Complement component C4B (C4)	K02403	**6**p21.33	1.4			
Complement protein component C7	J03507	**5**p13.1	− 2.5	− 2.7	− 2.6	− 2.7
Complement component C3, α- and β-subunits (C3)	K02765	**19**p13.3		− 2.5	− 2.5	− 3.1
**Cytoplasmic ribosomal proteins**						
Ribosomal protein L5	U14966	**1**p22.1			− 1.1	
Ribosomal protein S8	X67247	**1**p34.1			− 1.1	
Ribosomal protein S9	U14971	**19**p13.42			− 1.0	
Ribosomal protein S10	U14972	**6**p21.31			− 1.1	
**Cytoplasmic tRNA synthetases**						
Tryptophanyl-tRNA synthetase (TrpRS)	M61715	**14**q32.2	1.5	1.3	1.4	
**Eicosanoid synthesis**						
Prostacyclin (prostaglandin I2) synthase	AL118525	**20**q13.13	− 1.6	− 1.6		
Prostaglandin D2 synthase	BC005939	**9**q34.3	1.7		− 2.7	− 1.0
Phospholipase A2, membrane associated precursor	M22430	**1**p36.13	− 1.1			
Arachidonate 5-lipoxygenase	J03600	**10**q11.21	2.2			− 2.6
**Electron transport chain**						
Cytochrome bc-1 complex core protein II	J04973	**16**p12.2	− 1.0			
Uncoupling protein 2 (UCP2)	U94592	**11**q13.4	1.2	1.4		
Cytochrome c	BC005299	**7**p15.3		1.2		
Cytochrome c oxidase subunit VIIb (COX7b)	Z14244	**X**q21.1		1.1		
Cytochrome c oxidase copper chaperone (COX17)	L77701	**3**q13.33		1.0	1.1	
NADH-ubiquinone oxidoreductase AGGG subunit	AF050639	**7**q34		1.1		
ATP synthase lipid-binding protein	AL080089	**17**q21.32			1.2	
**Fatty acid degradation**						
Long-chain acyl-coenzyme A synthetase (FACL1)	L09229	**4**q35.1	− 1.0		− 1.1	
Carnitine palmitoyltransferase I, mithocondrial protein	U62733	**22**q13.33	1.3			
Long-chain fatty acid coA ligase 2	D10040	**4**q35.1			1.2	
**Gap junction proteins-connexins**						
Connexin 43	X52947	**6**q22.31	− 1.0			
Connexin 37	M96789	**1**p35.1	1.1			
Connexin 31	AF099730	**1**p34	1.3			
**Glycolisis and gluconeogenesis**						
Phosphoglycerate mutase	J04173	**10**q24.1			1.2	
**G-protein signaling**						
G protein β 5 subunit (Gβ5)	AF017656	**15**q21.2	− 1.7		− 1.4	
cAMP-specific phosphodiesterase	L20971	**1**p31.2	− 2.4			
cAMP-dependent protein kinase subunit RII-β (PRKARII β)	M31158	**7**q22.3	− 1.2		− 2.3	
Inositol 1,4,5-trisphosphate receptor type 1 (IP3R1)	D26070	**3**p26.1	− 2.0		2.1	
G-protein alpha subunit 14 (Gα14)	AF105201	**9**q21.2	− 1.7			
Protein kinase C β 2 (PKC β 2)	M18255	**16**p12.2			− 3.5	− 2.1
**GPCRs class A rhodopsin-like**						
Bradykinin receptor B2 (BDKRB2)	AF378542	**14**q32.2		1.5	2.7	
Thrombin receptor (F2R)	M62424	**5**q13.3		1.2	1.7	
Chemokine receptor-4 (CXCR4)	AJ224869	**2**q21.3		1.1	− 1.2	− 2.0
Encephalopsin (opsin3, panopsin)	AF140242	**1**q43		− 1.0		
G protein-coupled receptor V28 (chemokine receptor-1)	U20350	**3**p22.2	1.3			
Adenosine A1-receptor (ADORA1)	L22214	**1**q32.1	2.3			
C5a anaphylatoxin receptor (C5R1)	M62505	**19**q13.32			1.8	
Monocyte chemoattractant protein 1 receptor (MCP-1 R)	D29984	**3**p21.31			− 1.8	
Human Epstein-Barr virus-induced G-protein coupled receptor (EBI 1, CCR 7)	L31582	**17**q21.2			− 1.7	− 2.1
Neuropeptide Y receptor Y1 (NPYY1)	L07614	**4**q32.2				− 1.5
CB1 cannabinoid receptor (CNR1)	U73304	**6**q15				− 1.2
Apelin receptor	U03642	**11**q12.1			1.8	
Q9BXA0	AF348491	**2**q21.3			− 1.4	− 1.7
**GPCRs class B secretin-like**						
Lectomedin-1 γ	AF104939	**1**p31.1		− 1.0	− 1.4	
ETL protein	AF192403	**1**p31.1		1.0	1.3	
**GPCRs class C metabotropic glutamate**						
Putative G protein-coupled receptor (RAIG1)	AF095448	**12**p13.2	1.2	1.6		
GABA-B-R2	AF099033	**9**q22.33	2.6			
GPCR 5B protein	AC004131	**16**p12.3	1.1			
**GPCRs others**						
Duffy blood group antigen (FY)	U01839	**1**q23.1			− 1.6	− 1.6
**G13 signaling**						
GDP-dissociation inhibitor protein	L20688	**12**p12.3	1.4			
Phosphoinositide 3-kinase PI3-K D	Y10055	**1**p36.22				− 1.8
IQGAP2	Q13576	**5**q13.3			1.1	
**Inflammatory response**						
Cellular fibronectin precursor	X02761	**2**q35	3.4	1.2	2.6	
Laminin gamma2 chain	U31201	**1**q25.3	1.1			
Tumor necrosis factor receptor type 1	X55313	**12**p13.31	1.0			
Lymphocyte-specific protein-tyrosine kinase (LCK)	M21510	**1**p35.2			− 1.5	− 2.1
Interleukin 2 receptor γ chain	D11086	**X**q13.1			− 1.0	− 1.2
**MAPK cascade**						
GTPase-activating protein ras p21	M23379	**5**q14.3	1.0			
c-Jun	J04111	**1**p32.2			− 1.9	− 1.8
**Matrix metalloproteinases**						
Tissue inhibitor of metalloproteinase-3 (TIMP3)	U67195	**22**q12.3		1.2		
Collagenase type IV (MMP-2)	M58552	**16**q12.2	− 1.4			
Tissue inhibitor of metalloproteinases-1 (TIMP1)	X03124	**X**p11.3	2.0			
Matrix metalloproteinase 9 (MMP-9)	BC006093	**20**q13.12				− 1.6
**Nuclear receptors**						
β-glucocorticoid receptor	X03348	**5**q31.3		− 1.1		
Apolipoprotein AI regulatory protein	M64497	**15**q26.2		− 1.0		
Retinoid X receptor-γ	U38480	**1**q23.3	2.6			
**Nucleotide metabolism**						
NAD-dependent methylene tetrahydrofolate dehydrogenase cyclohydrolase (CH2H4 Folate DH)	X16396	**2**p13.1				− 1.0
**Peptide GPCRs**						
ATPaseII	AB013452	**4**p13			− 2.3	
Bradykinin receptor B2 (BDKRB2)	AF378542	**14**q32.2		1.5	2.7	
Monocyte chemoattractant protein 1 receptor (MCP-1 R)	D29984	**3**p21.31			− 1.8	
Human Epstein-Barr virus-induced G-protein coupled receptor (EBI 1, CCR 7)	L31582	**17**q21.2			− 1.7	− 2.1
Chemokine receptor-4 (CXCR4)	AJ224869	**2**q21.3		1.1	− 1.2	− 2.0
Duffy blood group antigen (FY)	U01839	**1**q23.1			− 1.6	− 1.6
C5a anaphylatoxin receptor (C5R1)	M62505	**19**q13.32			1.8	
Neurokinin 1 receptor (NKIR)	M76675	**2**p12			− 1.9	
Neuropeptide Y receptor Y1 (NPYY1)	L07614	**4**q32.2				− 1.5
G protein-coupled receptor V28 (chemokine receptor-1)	U20350	**3**p22.2	1.3			
**Proteasome degradation**						
Histone H2A.x	X14850	**11**q23.3	1.2			
Proteasome subunit LMP7	U17496	**6**p21.32	1.3			− 1.0
**Small ligand GPCRs**						
CB1 cannabinoid receptor (CNR1)	U73304	**6**q15				− 1.2
**TGF B signalling**						
Transforming growth factor beta 1 precursor (TGF-β1)	BC000125	**19**q13.2	1.4			
Transcription factor ISGF-3 (STAT1)	M97935	**2**q32.2	1.0			
Mad-related protein (SMAD1)	U54826	**4**q31.21	− 1.2			
KIAA0569 protein (Sip1)	AB011141	**2**q22.3	− 1.3			
TGF-β type III receptor	L07594	**1**p32.2			− 1.2	
SMAD4 (DPC4)	AF045438	**18**q21.1				1.1
c-Jun	J04111	**1**p32.2			− 1.9	− 1.8
Thrombospondin	J04835	**15**q15.1			2.2	
Lymphoid enhancer binding factor 1 (LEF-1)	AF198532	**4**q25			1.9	
**Translation factors**						
Eukaryotic translation initiation factor 2 α kinase 3	AF110146	**2**p11.2		1.7		
Eukaryotic translation initiation factor eIF-2 α subunit	L19161	**X**p22.11	− 1.3			
**Wnt signaling**						
Frizzled 1	AF072872	**7**q21.13	− 1.2		− 1.0	
Proto-oncogene (Wnt-5a)	L20861	**3**p14.3	1.0		− 1.4	
Cyclin D1	BC000076	**11**q13.3	1.5	1.4	1.4	
Cyclin D2	D13639	**12**p13.32	1.0			
Cyclin D3	M92287	**6**p21.1	1.3			
Urokinase-type plasminogen activator precursor	M15476	**10**q22.2	1.6			
Frizzled 7	AB017365	**2**q33.1		− 1.4		
Frizzled 10	AB027464	**12**q24.33		− 1.0		
c-Jun	J04111	**1**p32.2			− 1.9	− 1.8
Protein kinase C β 2 (PKC β 2)	M18255	**16**p12.2			− 3.5	− 2.1
Frizzled 2	AB017364	**17**q21.31				− 1.0

Several genes appear more than one time because associated with different biological pathways

Only the cyclin-dependent kinase inhibitor 1 (Cip1/CDKN1A) gene was up-regulated in both functioning and non-functioning nodules of the two patients, while Cip1, the complement component 1 β chain gene (C1qβ), the tryptophanyl-tRNA synthetase gene (TrpRS), the cellular fibronectin precursor gene and cyclin D1 were up-regulated in functioning nodules of the two patients (Table 2). Only Cip1 gene was up-regulated in non-functioning nodules of both patients. Cip1, TrpRS, uncoupling protein 2 gene (UCP2), putative G-protein coupled receptor gene (RAIG1), cellular fibronectin precursor gene and cyclin D1 gene were up-regulated in functioning and non-functioning nodules of patient #1. Again Cip1 gene was up-regulated in both nodules of patient #2 (Table 2).

### Down-regulated genes

The protein kinase C β 2 (PKCβ2) was the gene with the strongest decrease of expression (signal log ratio − 3.5) in non-functioning nodule of patient #2 (Table 2).

C1s and complement protein component C7 were down-regulated in both functioning and non-functioning nodules of the two patients, while genes down-regulated in functioning nodules of both patients were C1s, complement protein component C7, long-chain acyl-coenzyme A synthetase (FACL1), G-protein β 5 subunit (Gβ5), cAMP-dependent protein kinase subunit RII β (PRKARIIβ) and Frizzled 1. In the non-functioning nodules of the two patients the down-regulated genes were C1s, complement protein component C7 and complement component C3 α- and β-subunits (C3). C1s, complement protein component C7 gene and prostacyclin (prostaglandin I2) synthase gene were down-regulated in both nodules from patient #1, while many genes (IAP 1, c-Jun, C1s, complement protein component C7, C3, prostaglandin D2 synthase, protein kinase C β 2, chemokine receptor-4, human Epstein-Barr virus-induced G-protein coupled receptor, Q9BXA0, duffy blood group antigen, lymphocyte-specific protein-tyrosine kinase, interleukin 2 receptor γ chain) were down-regulated in both nodules from patient #2 (Table 2).

### Determination of mRNA levels by using real-time RT-PCR

To validate the microarray expression data with an independent method, we carried out real-time quantitative RT-PCR analysis for a sub-set of 12 genes (Table 3) in functioning, non-functioning and healthy tissues from each patient. The Affymetrix signal intensity data were substantially confirmed by real-time PCR data for the subset of genes (Table 3).

**Table 3:** Expression of mRNA levels of the sub-set of 12 genes determined by real-time PCR

	Patient #1			Patient #2		
	FN	NN	Normal thyroid	FN	NN	Normal thyroid
CDKN1A/Cip1 Cyclin-dependent kinase inhibitor 1	12.13 ± 0.91	4.79 ± 0.42	2.36 ± 0.25	1.18 ± 0.15	0.74 ± 0.07	0.32 ± 0.05
C1qβ Complement component 1, β-chain	7.80 ± 0.71	4.30 ± 0.33	4.15 ± 0.48	7.88 ± 0.79	2.08 ± 0.21	1.91 ± 0.22
C1s Complement subcomponent C1s, α- and β-chains	0.75 ± 0.081	1.32 ± 0.15	7.88 ± 0.81	1.58 ± 0.16	1.15 ± 0.15	2.94 ± 0.30
C7 Complement protein component C7	0.09 ± 0.01	0.17 ± 0.02	2.55 ± 0.30	0.53 ± 0.04	0.56 ± 0.04	3.08 ± 0.32
C3 Complement component C3, α- and β-subunits	2.87 ± 0.27	0.64 ± 0.07	5.54 ± 0.56	0.58 ± 0.08	0.26 ± 0.03	2.72 ± 0.51
WARS/TrpRS Tryptophanyl-tRNA synthetase	5.85 ± 0.60	3.06 ± 0.33	2.11 ± 0.19	3.52 ± 0.35	1.00 ± 0.18	0.91 ± 0.15
ACSL1/FACL1 Long-chain acyl-coenzyme A synthetase	0.82 ± 0.07	1.97 ± 0.11	1.89 ± 0.13	0.23 ± 0.06	1.00 ± 0.21	0.65 ± 0.08
GNβ5 G protein β 5 subunit	0.76 ± 0.08	1.17 ± 0.12	1.55 ± 0.17	0.35 ± 0.05	0.87 ± 0.09	0.88 ± 0.07
PRKARII β cAMP-dependent protein kinase subunit RII-β	1.82 ± 0.19	3.1 ± 0.25	3.83 ± 0.42	0.83 ± 0.09	2.01 ± 0.21	1.63 ± 0.17
FN1 Cellular fibronectin precursor	62.5 ± 6.81	9.54 ± 1.11	4.03 ± 0.47	4.51 ± 0.65	0.99 ± 0.09	0.66 ± 0.07
FZD1 Frizzled 1	4.71 ± 0.58	6.82 ± 0.71	7.48 ± 0.84	0.89 ± 0.08	2.14 ± 0.25	1.22 ± 0.17
CCND1 Cyclin D1	3.44 ± 0.35	2.32 ± 0.21	1.52 ± 0.16	1.97 ± 0.22	1.22 ± 0.18	0.98 ± 0.08

Results are expressed as pg RNA of the target gene/pg RNA of the housekeeping β-actin gene

The expression levels of the thyroid-specific genes thyrotropin receptor (TSHr), thyroid peroxidase (TPO), thyroglobulin (Tg) and sodium/iodide symporter (NIS) were not deregulated by the microarray analysis. By real-time PCR (Table 4), no significative differences in TSHr and Tg signals in functioning and non-functioning nodules with respect to the non-nodular thyroid tissue were shown, and an increase of TPO and NIS signals in functioning nodules were observed (Table 4). Real-time RT-PCR is commonly used to measure gene expression because it is also more sensitive than microarrays in detecting small changes in expression even if it requires more input RNA and is less adaptable to high-throughput studies [14]. This is probably the explanation for inconsistent results with respect to microarray expression data for NIS and TPO genes.

**Table 4:** Thyroid specific gene expression mRNA levels determined by real-time PCR in FNs and NNs of the two patients

	Patient #1			Patient #2		
	FN	NN	Normal thyroid	FN	NN	Normal thyroid
TSHr	1.25 ± 0.15	2.25 ± 0.21	2.66 ± 0.30	0.54 ± 0.06	1.31 ± 0.11	0.84 ± 0.07
TPO	12.2 ± 1.32	8.32 ± 0.71	1.03 ± 0.99	3.15 ± 0.35	1.68 ± 0.19	0.98 ± 0.09
Tg	1.65 ± 0.17	2.21 ± 0.22	1.44 ± 0.18	0.84 ± 0.07	0.72 ± 0.07	0.62 ± 0.05
NIS	3.75 ± 0.38	0.67 ± 0.07	0.50 ± 0.05	2.94 ± 0.31	0.16 ± 0.02	0.14 ± 0.02

Results are expressed as pg RNA of the target gene/pg RNA of the housekeeping gene

## Discussion

Autonomous or toxic nodular goiter is the most common form of hyperthyroidism in iodine deficiency areas where aged patients with long-standing non-toxic goiter experience a progressive increase in size and number of thyroid nodules [1, 2, 15]. With this process, thyroid function may progress from a fully TSH-dependent condition to autonomy and then to overt thyrotoxicosis [15]. In AMNG or TMNG most of the functioning thyroid nodules coexist with non-functioning ones [1, 2].

Non-functioning thyroid nodules are inactive and less differentiated with respect to functioning ones, and their molecular etiology is unknown. Moreover, malignant transformation is observed in nearly 5–10% of non-functioning thyroid nodules [16]. While functioning nodules growth is mediated by the activation of cAMP/PKA cascade [17] different metabolic signaling pathways may be implicated in the growth and the loss of the ability to trap iodine in non-functioning nodules [10].

Gene expression assays are able to identify thousands of unique characteristics for each tumor type and for this we decided to use this technology to compare the gene expression profile between functioning and non-functioning thyroid nodules with respect to healthy tissue arising in the same glands of two patients with autonomous or toxic nodular goiter coming from an area of iodine deficiency. The number of studied patients is certainly small, but significant for a preliminary and descriptive approach.

We demonstrated that the gene expression profile in both functioning and non-functioning thyroid nodules arising in the same thyroid gland was similar, and most of the modulated genes belonged to the same biological pathways. Nevertheless, in functioning nodules of both patients the total number of genes that changed their expression level (up-regulated and down-regulated genes) was greater with respect to the non-functioning ones.

Using the software GenMAPP we identified a total of 109 selected genes, located in 29 metabolic pathways, that increased or decreased at least 2-fold their expression level in nodular tissues. Most of the transcripts were down-regulated in functioning and non-functioning nodules compared with the surrounding tissue, but we focused our interest particularly on genes that were simultaneously up-regulated or down-regulated in both functioning or in both non-functioning nodules of the two patients.

In functioning nodules of both patients, an up-regulation of the cyclin D1 (BC000076) gene was observed. Cyclin D1 acts by complexing with cyclin-dependent kinase cdk4 or cdk6, promoting phosphorilation and inactivation of the tumor suppressor protein pRb and sequential events including the activation of E2F transcription factor. Overexpression of cyclin D1 contributes to the progression of the cell from G1 to S phase. Interestingly, the cyclin-dependent kinase inhibitor 1 (U09579) gene was up-regulated in the functioning and non-functioning nodules of both patients. We speculated that the simultaneous increase of Cip1 in both entities of the two patients very likely reflects a feedback control mechanism of cell proliferation. The product of Cip1 gene is p21 protein that may be the important intermediate by which p53 mediates its role as an inhibitor of cellular proliferation in response to DNA damage; it may bind to and inhibit cyclin-dependent kinase activity, preventing phosphorylation of critical cyclin-dependent kinase substrates and blocking cell cycle progression. A high expression of this protein may indicate a role of the p21/p53 pathway in the proliferation of thyroid nodules. Different reports described the overexpression of cyclin D1 in thyroid cancer. Wang et al. [18] found cyclin positivity in 63% of the follicular variant of papillary carcinoma and in 60% of follicular adenomas by using immunohistochemistry. An immunohistochemical positivity for p21/Cip1 protein has also been shown to be more frequent in well-differentiated thyroid carcinomas than in follicular adenomas [19].

Complement component C1s (M18767) and complement protein component C7 (J03507) genes were down-regulated in both functioning and non-functioning nodules suggesting a possible silencing of the non-specific immune response mediated by the complement activity. C1s combines with C1q to form C1, the first component of the classical pathway of the complement system, while C7 is a constituent of the membrane attack complex. Complement component C3 (K02765) gene was down-regulated in both non-functioning nodules and one functioning nodule of the two patients. This protein contains the C3a anaphylatoxin, a mediator of the local inflammatory process; it induces the contraction of smooth muscle, increases vascular permeability and causes histamine release from mast cells and basophilic leukocytes. Complement system activation has been demonstrated by immunohistochemistry and immunoelectron microscopy in thyroid carcinoma and in thyroid follicular adenomas [20]. It has been shown that complement can be activated by direct C4 binding to the CCP-like module of TPO without any mediation by Ig [21]. The significance of a reduction in complement activity in benign thyroid nodules remains unknown. Recently, an important down regulation of lymphocyte-specific genes in toxic adenomas [22] has been demonstrated using microarray analysis, and the presence of few lymphocytes in these nodules was also described [23]. The decreased complement components expression in functioning and non-functioning nodules likely extends this conclusion to macrophages.

The long-chain acyl-CoA synthetase (L09229) gene was down-regulated only in functioning nodules. This protein determines activation of long-chain fatty acids for both syntheses of cellular lipids and degradation via β-oxidation.

Functioning nodules of AMNG or TMNG are mostly due to chronic activation of the cAMP/PKA pathway [17], so it’s not amazing to find modifications in the expression of genes of G-protein signaling in these nodules. G protein β 5 subunit (AF017656) and cAMP-dependent protein kinase subunit RII-β (M31158) genes were down-regulated only in functioning nodules. The first protein is required for the GTPase activity, for replacement of GDP by GTP and for G-protein-effectors interaction, while the second one mediates membrane association by binding to anchoring proteins, including MAP2 kinase. A significant down-regulation of PKCβ2 was observed in both functioning and non-functioning nodules of patient #2. This finding is of particular interest in nodules characterized by increased proliferation and decreased activity. It has been shown that long-term stimulation of the PKC pathway with 12-*O*-tetradecanoyl-phobol-13-acetate in ovine, porcine and dog cultured thyroid cells causes a general loss of thyroid-specific functions. In particular, it was demonstrated that PKC inhibited TSH-mediated human thyroid cell differentiation [24].

Cellular fibronectin precursor (X02761) gene was up-regulated in functioning nodules of both patients. Fibronectins bind cell-surface proteins such as integrins and various intra and extracellular cell components including collagen, fibrin, heparin, DNA and actin. They are involved in cell adhesion, cell motility, opsonization, wound healing and maintenance of cell shape. The fibronectin gene has been found to be up-regulated in papillary thyroid carcinoma compared to normal thyroid [25]. Recently, Prasad et al. [26] demonstrated by immunohistochemistry that fibronectin expression is significantly associated with malignancy and is highly specific for carcinoma compared to adenoma [26]. The role of an increased expression of this gene in a benign pathology as the functioning thyroid nodule remains to be elucidated.

Frizzled 1 (AF072872) gene was down-regulated only in functioning nodules of both patients. Frizzled 7 (AB017365) gene and Frizzled 10 (AB027464) were down-regulated only in non-functioning nodules of patient #1. Recently, it has been demonstrated that elements of Wnt/beta-catenin signaling pathway are expressed in thyroid cells (nodular goiter and normal tissue adjacent to thyroid carcinoma) and are functionally active [27]. The highly conserved Wnt signaling pathway regulates cell proliferation, differentiation and cell fate and might play an important role in proliferation, differentiation and, when dysregulated, in thyroid tumorigenesis.

Summarizing, functioning and non-functioning thyroid nodules are basically tumors with increased proliferation rate and the functional hyperactivity of the functioning ones may be somehow a side characteristic of this specific kind of tumor. Although the activation of TSH receptor downstream effectors and target genes are supposed to be a very specific and prominent phenomenon, they first lead to the tumoral phenotype characterized by increased growth.

In conclusion, to our knowledge this is the first study that compares the gene expression profile in functioning and non-functioning thyroid nodules located within the same gland of patients affected by autonomous thyroid multinodular goiter from an area of iodine deficiency. The most surprising result emerged from this study is represented by the similar modulation in genes implicated in thyroid growth in different entities such as functioning and non-functioning nodules. Although obtained from a small number of patients, our results may represent a hint to understand the molecular pathogenesis of benign thyroid nodules. Further proteomic and metabolomic studies will be necessary to validate discoveries made at the genomic level.
